# Leptin changes differentiation fate and induces senescence in chondrogenic progenitor cells

**DOI:** 10.1038/cddis.2016.68

**Published:** 2016-04-14

**Authors:** X Zhao, Y Dong, J Zhang, D Li, G Hu, J Yao, Y Li, P Huang, M Zhang, J Zhang, Z Huang, Y Zhang, Y Miao, Q Xu, H Li

**Affiliations:** 1 Department of Orthopaedics, Ren Ji Hospital, School of Medicine, Shanghai Jiao Tong University, Shanghai, China; 2 Department of Joint Surgery, The First People's Hospital of Lianyungang, Lianyungang, China; 3 Department of Transplantation and Hepatic Surgery, Ren Ji Hospital, School of Medicine, Shanghai Jiao Tong University, Shanghai, China; 4 Department of Animal Facility, Ren Ji Hospital, School of Medicine, Shanghai Jiao Tong University, Shanghai, China; 5 Ren Ji-Med X Stem Cell Research Centre, Ren Ji Hospital, School of Medicine, Shanghai Jiao Tong University, Shanghai, China; 6 Department of Cardiology, Ren Ji Hospital, School of Medicine, Shanghai Jiao Tong University, Shanghai, China; 7 Department of Orthopaedics, Xinhua Hospital, School of Medicine, Shanghai Jiao Tong University, Shanghai, China; 8 Traditional Chinese Medicine Department, Ren Ji Hospital, School of Medicine, Shanghai JiaoTong University, 160 Pujian Road, Shanghai 200127, China

## Abstract

Body weight is a component of the mechanical theory of OA (osteoarthritis) pathogenesis. Obesity was also found to be a risk factor for digital OA involving non-weight-bearing joints, which suggested that metabolism influences the occurrence and progression of OA. The metabolic origin of OA has been partially attributed to the involvement of adipokines, such as leptin, the levels of which are significantly and positively correlated with cartilage degeneration in OA patients. However, the mechanisms by which leptin-induced cartilage degeneration occurs are poorly understood. The discovery of chondrogenic progenitor cells (CPCs) opened up new opportunities for investigation. Investigating the effects of leptin on differentiation and proliferation in CPCs would increase our understanding of the roles played by leptin in the aetiology and development of OA. Here, CPCs were harvested using single-cell sorting from rat cartilage tissues to obtain mesenchymal stem-like cells, which possess clonogenicity, proliferation and stemness. High doses of leptin decreased the ability of the CPCs to migrate, inhibited their chondrogenic potential and increased their osteogenic potential, suggesting that leptin changes differentiation fates in CPCs. High doses of leptin induced cell cycle arrest and senescence in CPCs by activating the p53/p21 pathway and inhibiting the Sirt1 pathway. Inhibiting the Sirt1 pathway accelerated cartilage senescence in knockout (KO) mice. Activating the leptin pathway induced higher Ob-Rb expression and was significantly correlated with cartilage degeneration (lower levels of Coll-2) and tissue senescence (higher levels of p53/p21 and lower levels of Sirt1) in OA patients, suggesting that leptin-induced CPCs senescence contributes to the development of OA. Taken together, our results reveal new links between obesity and cartilage damage that are induced by leptin-mediated effects on cell behaviour and senescence.

Chondrogenic progenitor cells (CPCs) as cartilage seed cells are important to maintain cartilage homeostasis.^1, 2^ CPCs were first identified in calf cartilage as a subpopulation of superficial zone cells that were found to be required for appositional growth in articular cartilage.^3^ Koelling *et al.*^1^ also found CPCs in articular cartilage during the later stages of human osteoarthritis (OA). These progenitor cells migrate towards local injury sites, where they proliferate and differentiate as needed to replace damaged tissue.^2^ Mesenchymal stem progenitor cells extensively expand and can differentiate into multiple lineages, including bone, cartilage, tendon, muscle and nerve cells, under specific culture conditions *in vitro.*^4, 5^ A decline in muscle stem cell function was suggested as a potential underlying cause for the reduced ability of aging mammals to maintain skeletal muscle tissue.^4^ The same effect was observed in cartilage tissue. Inhibiting the functions of CPCs may therefore be a risk factor for OA.

We previously found that inflammatory mediators of OA that are found in the synovial fluid induced the degeneration of normal articular cartilage when injected into the intact joint cavities of beagle dogs.^6^ Among these inflammatory cytokines, leptin has received particular interest because of its role in influencing body weight homeostasis and the significant and positive correlation between leptin and cartilage degeneration.^7^ The leptin that is produced by white adipose tissue is a small (16 kDa), non-glycosylated protein that is encoded by the *Ob* gene.^8^ Leptin and its receptor have been isolated from human chondrocytes, osteophytes, synovium and infrapatellar fat pads.^9, 10^

Stannus OP *et al.* provided evidence showing that serum levels of leptin are independently and consistently associated with reduced cartilage thickness both cross-sectionally and longitudinally, suggesting that leptin plays an important role in the aetiology and development of OA.^8^ Simopoulou *et al.*^11^ also found that the expression of leptin and the leptin receptor (Ob-Rb) was significantly higher in the cartilage of patients with advanced OA than in the cartilage of patients with mild OA. Moreover, the proteoglycans in articular cartilage were depleted when leptin was injected into the stifle joints of rats.^10^ Previous studies investigated the effects of leptin on the biological functions of chondrocytes.^12^ However, the mechanisms that mediate leptin-induced cartilage degeneration are not well-defined. Investigating the effects of leptin on the biological functions of CPCs would increase our understanding of the roles that leptin plays in cartilage degradation.

Here, we isolated and identified CPCs from the cartilage of the stifle joints of rats. Leptin changed the differentiation fate of CPCs by inhibiting chondrogenesis and increasing osteogenesis. Leptin also induced senescence in CPCs by activating the p53/p21 pathway and inhibiting the Sirt1 pathway. The activation of the leptin pathway (higher levels of Ob-Rb) was significantly correlated with cartilage degeneration (lower levels of Coll-2) and tissue senescence (higher levels of p53/p21 and lower levels of Sirt1) in OA. Taken together, our results reveal new links between obesity and cartilage damage that are induced by leptin-mediated effects on cell behaviour, senescence and intracellular signalling.

## Results

### Identification of CPCs isolated from cartilage

**Figure 1:**
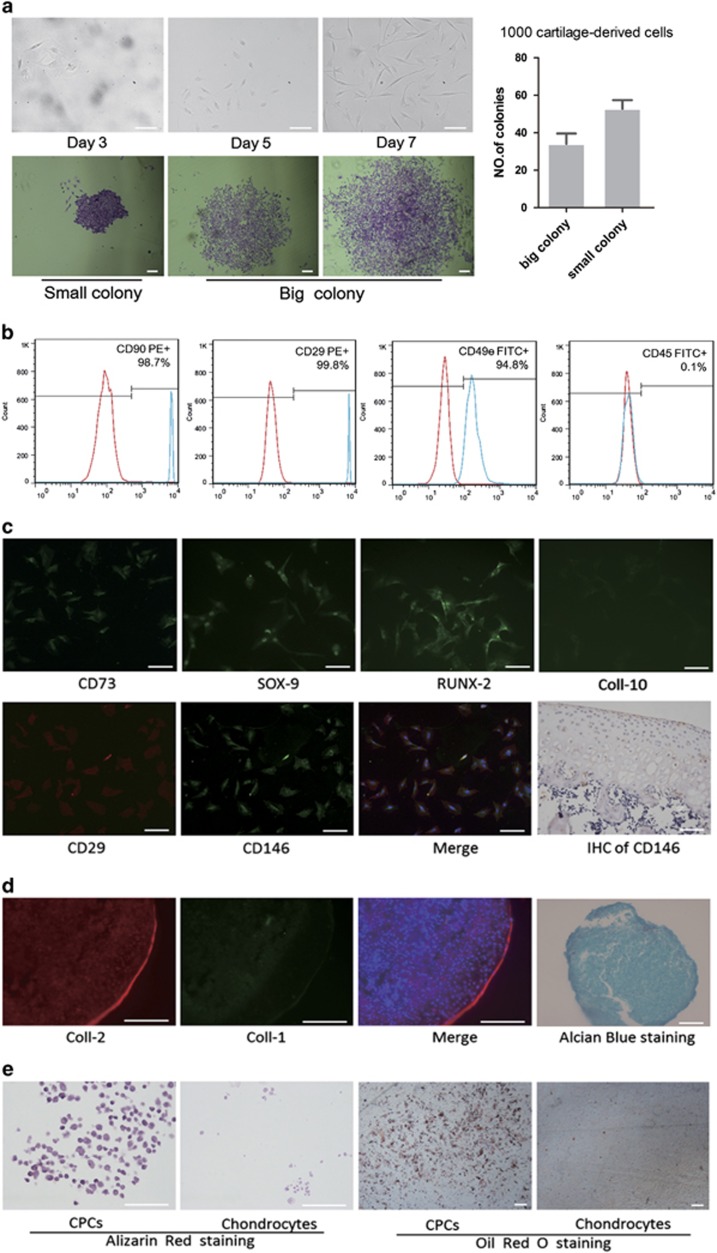
Isolation and characterization of and multipotent differentiation ability in CPCs. (**a**) Morphological characteristics are shown for colonies obtained from full-thickness cartilage after cells were cultured for 3, 5 or 7 days (magnification, × 200). The colonies were stained with crystal violet, and the colony-forming efficiency of the rat cartilage-derived cells was analysed on day 10. The results are shown as the mean±S.E.M. (**b**) Flow cytometric analysis of CPCs surface markers. Note that stem cell-relevant markers, such as CD29, CD90 and CD49e, are positive but that haematopoietic markers, such as CD45, are negative. (**c**) Immunofluorescence results for marker proteins (CD73, CD146, SOX-9, RUNX-2 and Coll-10) that were used to identify and characterize the colonies (magnification, × 200). The results of immunocytochemistry revealed that CD146-positive cells are primarily distributed throughout the surface layer in the knee tissues of 8-week-old SD rats. The multidifferentiation potential of CPCs *in vitro*. (**c**) Pellets of CPCs were assessed using double-labelled immunofluorescence for Coll-2 and Coll-1 (magnification, × 400) and Alcian Blue staining (magnification, × 200). (**e**) Alizarin Red S staining was used to evaluate osteogenic differentiation in CPCs and chondrocytes (magnification, × 400). (Oil red O staining was used to evaluate adipogenic differentiation in CPCs and chondrocytes (magnification, × 40). Scale bar, 100 *μ*m

The criteria that are commonly used to define stem/progenitor cells are clonogenicity, multipotency and self-renewal.^13^ To isolate CPCs, which are cartilage-derived cells with clonogenicity, multipotency and self-renewal capacity, we generated and cultured single-cell suspensions from cartilage tissues obtained from the stifle joints of SD rats using a monoclonal method. A portion of the cartilage-derived cells was attached to the plate and remained quiescent for 1–2 days before they began to rapidly divide and form colonies. After 5–7 days, colonies had formed from single cells (Figure 1a). A small population (about 8–9%) of the rat cartilage-derived cells had formed adherent cell colonies by day 10 (Figure 1a). These colonies were heterogeneous in size and cell density, which potentially reflects differences in cell proliferation rates. We used flow cytometric analysis to examine the presence of surface antigens on the CPCs. Over 94% of cells (passage 1) were positive for the mesenchymal stem cell markers CD29, CD49e or CD90.1 and negative for the leukocyte marker CD45^13^ (Figure 1b). Moreover, most of the cells expressed specific markers of CPCs, including CD73 and CD146^1, 14^ (Figure 1c), the cartilage-specific transcription factor SOX-9,^1^ and RUNX-2, which is an important transcription factor during bone and cartilage development. The cells did not express Coll-10 (Figure 1c). We used a novel stem cell marker, CD146, to tag the CPCs for immunohistochemical staining, and we found that the CPCs were primarily distributed throughout the surface layer (Figure 1c). The CPCs underwent chondrogenic, osteogenic or adipogenic induction and were observed over a 21-day culture period to evaluate their multilineage differentiation potential. Pellets from the differentiated chondrocytes displayed substantial Coll-2 (type II collagen) deposition (Figure 1d) and did not express Coll-1 (type I collagen) on their surfaces (Figure 1d), as visualized in Coll-2 and Coll-1 immunofluorescence. The pellets that were assessed using Alcian Blue showed higher expression of glycosaminoglycan (Figure 1d). The results suggested that CPCs can differentiate into hyaline cartilage instead of fibrocartilage. Similarly, CPCs cultured in osteogenic medium exhibited more calcium phosphate deposition in the extracellular matrix than chondrocytes, as detected by Alizarin Red staining (Figure 1e). However, approximately half of the CPCs were positive for Oil Red O staining after adipogenic induction (Figure 1e).^2^ Thus, the colonies grown from cells with a primary mesenchymal progenitor phenotype that were isolated from cartilage displayed multipotent differentiation ability, and specific markers CPCs can be used to characterize CPCs.

### High doses of leptin decrease the migratory ability, inhibit the chondrogenic potential and increase the osteogenic potential of CPCs

**Figure 2:**
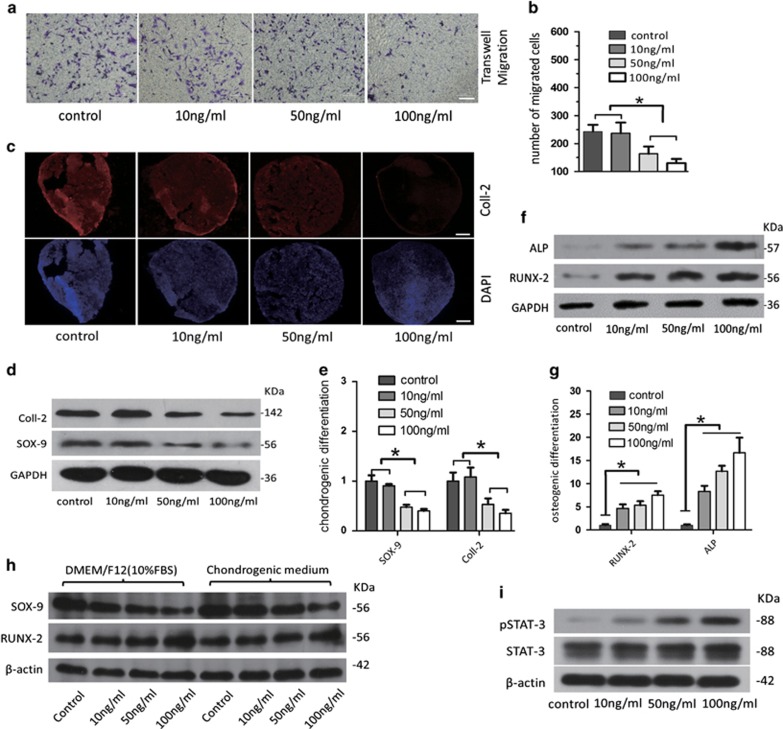
Changes in migration, chondrogenic potential and osteogenic potential were analysed in CPCs that were treated with different doses of leptin. (**a**) CPCs were treated with different doses of leptin (0 ng/ml as the control or 10, 50  or 100 ng/ml) while growing in culture medium. At 24 h after treatment was begun, the migrated CPCs were stained using crystal violet, and the numbers of CPCs were counted and averaged in five visual fields. (**b**) A lower number of migrated CPCs was observed in the cells treated with a high dose of leptin than in the control cells. (**c**) CPCs were grown in a chondrogenic induction system in the presence of leptin (0 ng/ml as the control or 10 , 50 or 100 ng/ml) for 21 days. Pellets were analysed using Coll-2 immunofluorescence and western blot analysis. (**d** and **e**) Western blot was performed to analyse the protein level of Coll-2 and SOX-9 in CPCs after 21 days of chondrogenic induction in the presence of leptin (0 ng/ml as the control or 10 , 50 or 100 ng/ml) *in vitro*. Relative protein abundance of each blot was normalized to the grey value of GAPDH (**f** and **g**). CPCs were grown in an osteogenic induction system in the presence of leptin (0 ng/ml as the control or 10, 50  or 100 ) for 7 days. Alkaline phosphatase (ALP) and RUNX-2 were detected using western blot analysis. Relative protein abundance of each blot was normalized to the grey value of GAPDH. ALP levels and the expression of the osteogenic transcription factor RUNX-2 were higher in the cells that were incubated with leptin than in the control cells. (**h**) The effect of a short duration of exposure to leptin treatment on CPCs cells was evaluated. SOX-9 and RUNX-2 expression were detected in CPCs grown in the presence of leptin (0 ng/ml as the control or 10 , 50 or 100 ng/ml) for 2 days. (**i**) After 2 days, leptin intracellular signalling (pSTAT-3) was activated at higher levels in CPCs that were treated with high doses of leptin than in the control cells or the cells treated with low doses of leptin. Scale bar, 100* μ*m. Error bars represent the mean±S.D. *P<0.05 was considered statistically significant

Normal CPCs functions, including migration and proliferation, in addition to their chondrogenic potential, play important roles in maintaining cartilage tissue homeostasis.^1^ We investigated the effects of leptin on the ability of CPCs to migrate and their chondrogenic potential to study the influence of leptin on these functions. A physiological dose of leptin in rats that is similar to a normal dose in humans is approximately 10 ng/ml, while a dose between 50  and 100 ng/ml is pathological in OA patients.^15, 16^ We therefore treated CPCs with the following doses of leptin: 0 ng/ml was used as a control, 10 ng/ml was used as a physiological dose and 50  and 100 ng/ml were used as high doses. We assessed the effects of each dose of leptin on CPCs migratory activity using Transwell assays. Treating cells with 50  and 100 ng/ml of leptin resulted in a lower number of migrated CPCs than was observed in the controls, suggesting that high doses of leptin inhibited migration in CPCs (Figures 2a and b). We next used pellet assays to examine the effect of leptin on cartilage formation and chondrogenic differentiation in cells grown in chondrogenesis-inducing medium for 21 days. Coll-2 expression (as Coll-2 immunofluorescence under the same exposure intensity) was significantly lower in the CPCs that were treated with high doses of leptin than in the control cells (Figure 2c). The expression levels of Coll-2 and SOX-9, a Coll-2 transcription factor, were also lower in the cells that were treated with 50  and 100 ng/ml leptin than in the control cells according to western blot analysis (Figures 2d and e), suggesting that high doses of leptin inhibit chondrogenic differentiation in CPCs. We also examined the impact of leptin on osteogenic differentiation in CPCs. After cells were cultured in osteogenic-inducing medium for 7 days, we found that the cells that were also treated with physiological and high doses of leptin showed a gradual increase in the expression of alkaline phosphatase, a key osteogenic enzyme, and the osteogenic transcription factor RUNX-2, whereas the control cells did not (Figures 2f and g). Leptin therefore significantly enhanced the osteogenic potential of cultured CPCs. We also evaluated the effects of a short duration of exposure to leptin on CPCs cells. Unlike the control and low doses of leptin, high doses of leptin activated leptin intracellular signalling (pSTAT-3)^17, 18^ in CPCs (Figure 2i), and only the highest dose of leptin (100 ng/ml) reduced the expression of SOX-9 and increased the expression of RUNX-2 in CPCs that were cultured in either normal medium (Dulbecco's modified Eagle's medium (DMEM)/F12 with 10% fetal bovine serum (FBS)) or chondrogenic medium for 2 days (Figure 2h). When grown while exposed to high doses of leptin, CPC functions known to be involved in maintaining cartilage homeostasis were reduced, and CPCs tended to undergo osteogenic differentiation.

### Leptin induces cell cycle arrest and senescence in CPCs

**Figure 3:**
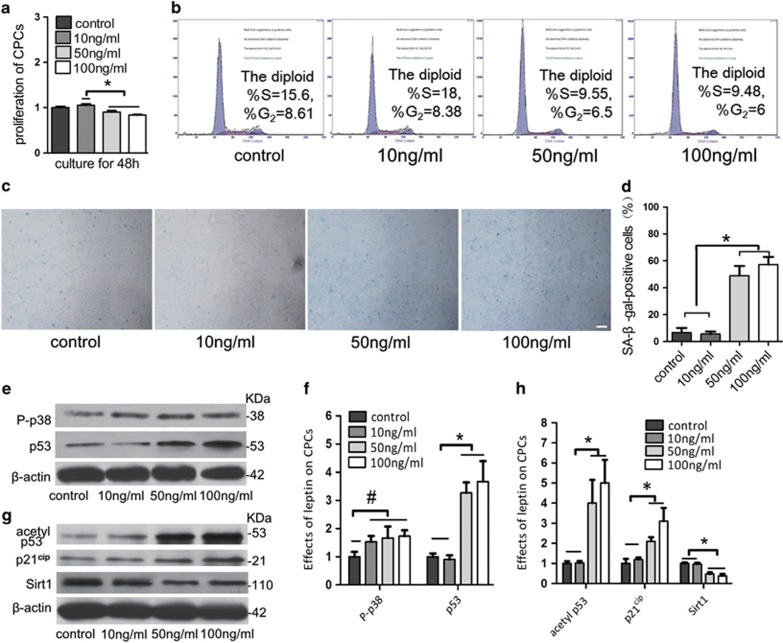
The activation of the p53/p21 pathway and the inhibition of the Sirt1 pathway promoted cell cycle arrest and senescence in CPCs that were treated with high doses of leptin. (**a**) CPCs were treated with leptin (0 ng/ml as the control or 10, 50 or 100 ng/ml) for 48 h. Compared with the control, high doses of leptin inhibited the proliferation of CPCs by CCK-8 assay. (**b**) Flow cytometry verified that the high doses of leptin induce CPCs cycle arrest by inhibiting the G_1_–S phase. (**c** and **d**) Staining for cell senescence. CPCs were treated with leptin (0 ng/ml as control, 10, 50  and 100 ng/ml) for 48 h, and the cells were then observed using light microscopy (× 40). Scale bar, 100 *μ*m. The percentage of CPCs that positively stained for SA-*β*-Gal was counted in five visual fields. High doses of leptin induced senescence in CPCs. (**e** and **f**) P-p38 and p53 expression were detected in CPCs grown in the presence of leptin (0 ng/ml as the control or 10, 50 or 100 ng/ml) for 2 days. Relative protein abundance of each blot was normalized to the grey value of *β*-actin. (**g** and **h**) Acetylp53, p21 and Sirt1 expression were detected in CPCs grown in the presence of leptin (0 ng/ml as the control or 10, 50 or 100 ng/ml) for 2 days. Relative protein abundance of each blot was normalized to the grey value of *β*-actin. Western blot analysis verified that the p53/p21 pathway was significantly activated and that the Sirt1 pathway was inhibited in CPCs that were treated with high doses of leptin. Error bars represent the mean±S.D. *P<0.05 was considered statistically significant. ^#^*P*>0.05 indicates that the results were not statistically significant

We next explored the effects of different doses of leptin (0 , 10, 50 and 100 ng/ml) on CPCs proliferation using CCK-8 assays and cell cycle analyses. Treating the cells with high doses of leptin resulted in less proliferation than was observed when cells were treated with control and physiological doses and also induced CPCs cell cycle arrest by inhibiting the G_1_–S cycle (Figures 3a and b). Cell cycle arrest generally leads to quiescence or senescence.^19^ Treating cells with 50 and 100 ng/ml leptin resulted in a higher percentage of SA-*β*-Gal-positive CPCs than were observed in the cells treated with control and physiological doses of leptin (Figures 3c and d). High doses of leptin therefore induce senescence in CPCs.

### Leptin increases the levels of p53, acetylated p53, p21 and Sirt1 during CPCs senescence

Two major pathways lead to the induction of cellular senescence: the p38 mitogen-activated protein kinase (MAPK)/p16^INK4a^ pathway and the p53/p21^cip^ pathway.^20^ The levels of phosphorylated p38 MAPK in CPCs were slightly higher in cells grown in the presence of physiological and high doses of leptin than in the control cells. However, the levels of p53, acetylated p53 and p21 were significantly higher in cells treated with high doses of leptin than in the control. Lower levels of the p53 deacetylase Sirt1 are often associated with p53-mediated senescence.^20^ The expression of Sirt1 was significantly lower in the cells that were treated with high doses of leptin than in the control cells, and acetylated p53 levels were also higher in these cells. Leptin may induce senescence in CPCs by activating the p53/p21 pathway and inhibiting Sirt1.

### Leptin induces senescence in CPCs via activation of the p53/p21 pathway

**Figure 4:**
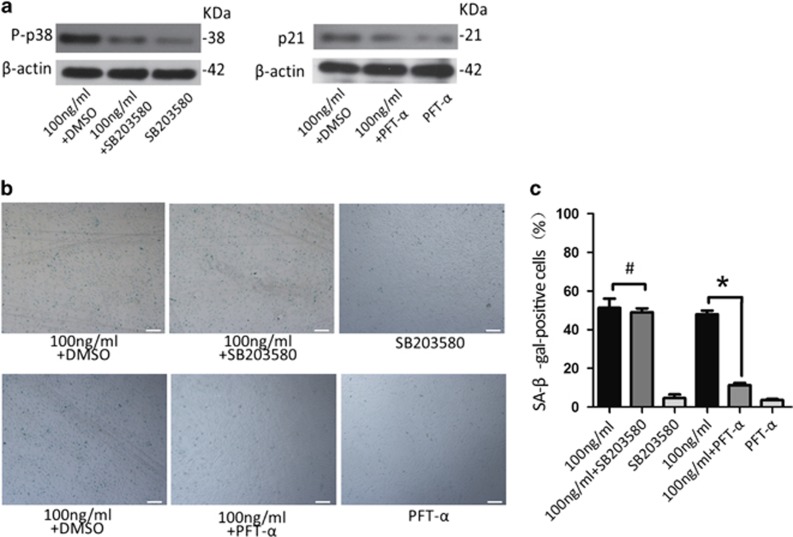
Blocking the p53/p21 pathway inhibits senescence in CPCs treated with leptin. (**a**) The p38 inhibitor SB203580 (10* μ*M) significantly decreased the expression of P-p38. The p53 inhibitor PFT-*α* (20 *μ*M) significantly decreased the expression of p21. (**b** and **c**) The percentage of SA-*β*-Gal-positive cells was significantly decreased after cells were treated with PFT-*α* and 100 ng/ml leptin but not after cells were treat with SB203580 and 100 ng/ml leptin for 48 h. Error bars represent the mean±S.D. Scale bar, 100 *μ*m. *P<0.05 was considered statistically significant. ^#^*P*>0.05 indicates that the results were not statistically significant

We performed a series of pathway inhibition tests to further explore the role of the p53/p21 pathway in CPCs senescence. CPCs were treated with a high dose of leptin and either the p38 inhibitor SB203580 or the p53 inhibitor PFT-*α*, and the effects of their inhibition were analysed (Figure 4a). Cell senescence was evaluated by determining the percentage of SA-*β*-Gal-positive cells. The p38 inhibitor SB203580 had no obvious effect on the percentage of SA-*β*-Gal-positive cells, but cells that were treated with the p53 inhibitor PFT-*α* displayed a significantly lower percentage of SA-*β*-Gal-stained cells (Figures 4b and c). Both the p53 and the P-p38 inhibitors failed to increase the percentage of senescent cells. These results indicate that leptin induces senescence in CPCs by activating the p53/p21 pathway.

### Induction of CPCs senescence by leptin via inhibition of the Sirt1 21/p53 pathway

**Figure 5:**
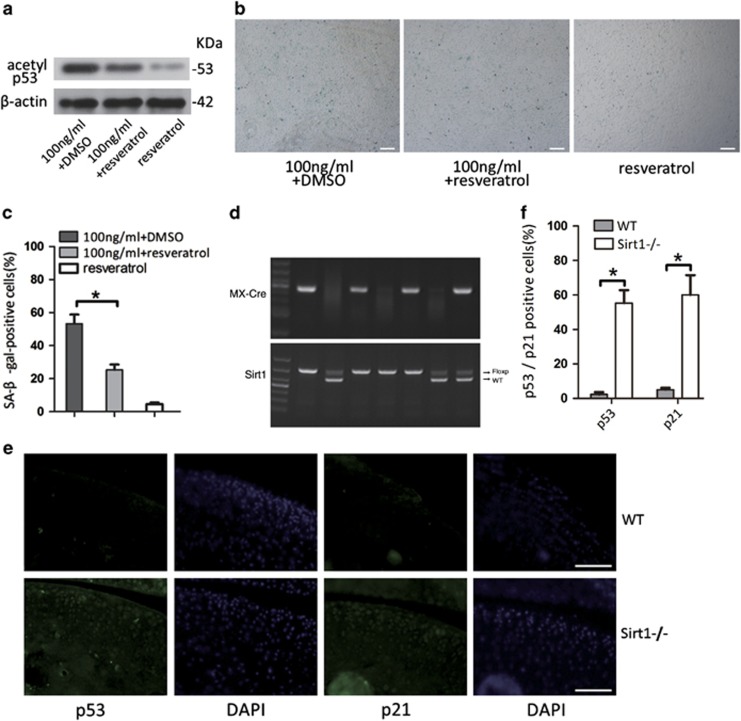
Activating and blocking Sirt1 affected CPCs senescence via the p53/p21 pathway induced by leptin. (**a** and **b**) Cells were treated with resveratrol and leptin, and the percentage of SA-*β*-Gal-positive cells was analysed. Cultured CPCs were treated with resveratrol (30 *μ*M), a Sirt1 pathway agonist, and 100 ng/ml leptin. (**c**) After 48 h of treatment, western blot analysis showed that acetylated p53 expression was inhibited. (**d**) PCR analysis of the genomic Sirt1 locus in heterozygous floxed mice with and without Cre and with or without tamoxifen. (**e** and **f**) Comparison of the number of p53- and p21-positive cells in wild-type and KO mice. Fluorescence microscopy images show that the p53/p21 pathway was more activated in the articular cartilage tissues of Sirt1 KO mice than in wild-type mice. Scale bar, 100 *μ*m. Error bars indicate the mean±S.D. *P<0.05 was considered statistically significant

To confirm that the Sirt1 pathway is involved in leptin-induced CPCs senescence, we investigated the effect of activating and blocking Sirt1. We found that when cells were treated with a high dose of leptin (100 ng/ml) and resveratrol (30 *μ*M), a Sirt1 activator, the expression of acetyl p53 was lower than in the control cells and that the percentage of SA-*β*-Gal-positive cells dropped to 25% (Figures 5b and c). This result indicates that activating Sirt1 prevents leptin-induced senescence in CPCs and that leptin induces senescence by inhibiting the Sirt1 pathway. To explore whether the leptin-induced inhibition of Sirt1 promotes senescence in cartilage tissues, we prepared articular cartilage tissue from the stifles of three-month-old Sirt1 KO (Sirt1−/−) mice (one month after knockout) to examine the effect of Sirt1 KO on p53 and p21 expression. Sirt1−/− mice exhibited significantly higher levels of p53/p21 than their wild-type littermates in cartilage tissue sections (Figures 5e and f). These results confirm that leptin induces senescence in CPCs by inhibiting the Sirt1 pathway and activating the p53/p21 pathway, which may disturb the maintenance of cartilage tissue homeostasis and lead to cartilage senescence.

### Activation of the p53/p21 pathway and inhibition of the Sirt1 pathway are independent of leptin levels and related to the function of Ob-Rb

**Figure 6:**
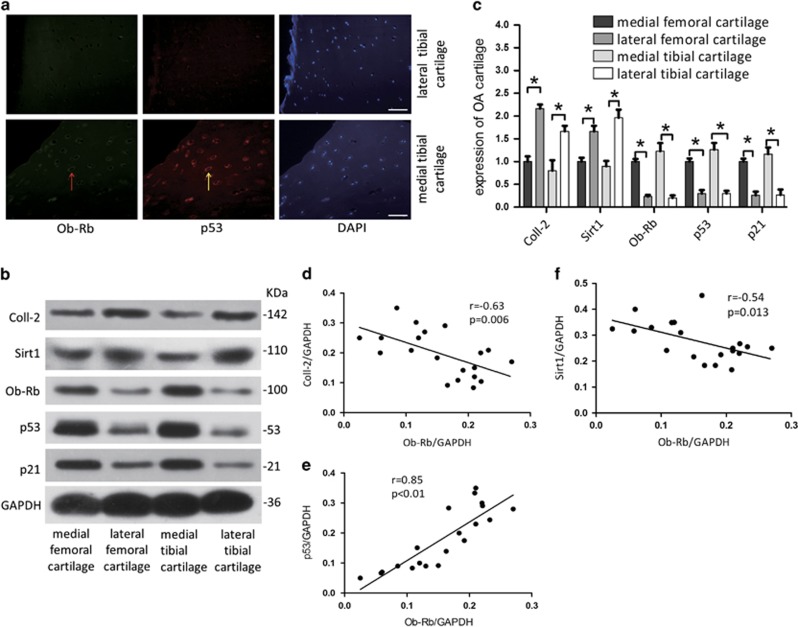
Changes in leptin-related signalling pathways were correlated with the severity of cartilage degeneration. (**a**) p53 and Ob-Rb double-labelling (200-fold magnification) in LTP and MTP cartilage (indicated by an arrow). Scale bar, 100 *μ*m. (**b**) Cartilage tissue proteins were extracted from the weight-bearing areas of the LFC, MFC, LTP and MTP in OA patients. Protein western blot analysis was used to detect the protein levels of Coll-2, Sirt1, Ob-Rb, p53 and p21 in those cartilage tissue proteins. (**c**) Relative protein abundance of each blot was normalized to the grey value of GAPDH. (**d–f**) The relationships between the expression levels of Ob-Rb and Coll-2, p53 and Sirt1 were analysed in five samples. Error bars represent the mean±S.D. *P<0.05 was considered statistically significant

Previous studies have collectively suggested that there is a significant positive correlation between leptin levels and the severity of cartilage degeneration. However, the degeneration of cartilage is generally unbalanced and asymmetrical within each OA patient, and medial cartilage lesions are generally more severe than lateral cartilage lesions in patients with the same leptin levels in both tissues. Leptin-induced CPCs senescence may therefore lead to cartilage degeneration over time, even in OA. We collected cartilage samples from OA patients to explore this possibility. To exclude the effect of differences in leptin levels between samples, we investigated cartilage tissues that were harvested from the weight-bearing femoral condyle (FC) and tibial plateau (TP) areas in the same OA knee joint of each patient. The cartilage lesions at the medial FC (MFC) and medial TP (MTP) were generally more severe than those in the lateral FC (LFC) and lateral TP (LTP) in OA joints. There was also more serious degeneration in the cartilage tissues obtained from the MFC and MTP that expressed lower levels of Coll-2 and Sirt1 and higher levels of Ob-Rb, p53 and p21 (Figures 6b and c). Immunofluorescence showed that MTP cartilage exhibited higher levels of p53 and Ob-Rb, whereas LTP cartilage exhibited lower levels of p53 and Ob-Rb (Figure 6a). As the expression of Ob-Rb increased, Coll-2 expression (*r*=−0.63, *P*=0.006) (Figure 6d) and Sirt1 expression (*r*=−0.54, *P*=0.013) significantly decreased (Figure 6f) while p53 expression significantly increased (*r*=0.85, *P*<0.01) (Figure 6e). We conclude that during leptin-induced senescence in CPCs, the constant degeneration of cartilage that is caused by the activation of the p53/p21 pathway and the inhibition of the Sirt1 pathway may be more dependent on the function of Ob-Rb than on leptin.

## Discussion

We found that articular cartilage contains a cell population that is characterized by a primary mesenchymal progenitor phenotype (Figures 1a and b), and this phenotype was maintained during expansion *in vitro*, consistent with the findings of previous CPCs studies.^1, 5^ We established CPCs cultures by using single-cell sorting to select cells with clonogenic potential. This approach enabled us to isolate a clonal population that represented CPCs features. We found that CPCs can be readily induced towards chondrogenic (differentiating into hyaline cartilage rather than to fibrocartilage) and osteogenic differentiation and that they can be induced to exhibit adipogenic ability (Figures 1d and e). However, other researchers have shown that CPCs possess very limited adipogenic ability.^5^ We suspect that the reason for this discrepancy is that we used cartilage that was obtained from rats that were going through puberty because the tissue in these animals exhibits a stronger stem-like character.

We used a specific marker, CD146, to label CPCs,^14, 21^ and we found that progenitor cells were primarily distributed throughout the surface layer (Figure 1c). This result is consistent with the findings of previous studies.^3^ These results suggest that CPCs may reside within the articular cartilage matrix until they are activated by certain conditions, such as trauma and inflammation, which require the damaged cartilage tissues to be restored.^5^

We therefore focused on the impact of leptin on CPCs functions, including their migratory and proliferative potential and their ability to undergo chondrogenic and osteogenic differentiation.^1^ We found that CPCs migration was reduced by high doses of leptin (50 and 100 ng/ml) (Figures 2a and b). Migration is an important characteristic of CPCs, particularly during their role in damage repair, suggesting that leptin inhibits the CPCs response during cartilage recovery following an injury. Our results also show that high doses of leptin inhibited the chondrogenic differentiation potential of CPCs when cells were grown in chondrogenesis-inducing medium (Figures 2c–e). This observation helps to explain why articular cartilage degeneration was induced in animal models after leptin was injected into the articular cavity.^10^ High doses of leptin enhanced the osteogenic potential in CPCs grown in osteogenesis-inducing medium (Figures 2f and g). This phenomenon resembles the leptin-induced increase in MSC osteogenic potential and bone formation^22, 23^ and may be important to OA treatment procedures, such as cartilage thinning, tidemark ante-displacement and perichondral ossification. We hypothesize that high doses of leptin alter the fate of differentiating CPCs. CPCs appear unable to undergo timely migration and differentiation into normal mature chondrocytes, which are required to repair damaged cartilage, and this leads to disequilibrium in cartilage tissue homeostasis and, over time, cartilage degeneration. This finding also explains the decreases that have been observed in ColI-2, SOX-9 and GAG (the main component of articular cartilage) levels and the increases that have been observed in Coll-1 (mainly in bone tissues) and Coll-X (chondrocyte hypertrophic differentiation)^24^ in OA cartilage tissues.^25, 26^ These changes destroy the cartilage tissue structure and result in cartilage that functions as an ineffective buffer.^26^

Furthermore, our investigations demonstrated that high doses of leptin inhibited the proliferation (Figures 3a and b) and induced cell cycle arrest in CPCs. Cell cycle arrest generally leads to quiescence or senescence.^19^ Quiescence is a reversible process that leads to a transient state of cell dormancy. In contrast, senescence results in an irreversible change in normal cell activities. Cell senescence was first observed in aged cells but can also occur in young cells. Senescent cells continue to grow and appear larger than their non-senescent counterparts.^27^ Senescence-associated hypertrophy leads to increases in the number and activity of lysosomes, a feature that is exploited in one of the most widely used assays to identify senescence, SA-*β*-Gal, which is based on lysosomal *β*-galactosidase activity.^15, 28^

We observed an increase in the percentage of SA-*β*-Gal-positive CPCs when cells were grown in the presence of high doses of leptin *in vitro* (Figures 3c and d). These data indicate that leptin induces senescence in CPCs. Two major pathways lead to the induction of cellular senescence: the p38 mitogen-activated protein kinase (MAPK)/p16^INK4a^ pathway and the p53/p21^cip^ pathway.^20^ We show that p53, acetylated p53 and p21 levels were significantly higher in leptin-treated CPCs than in the control CPCs (Figures 3e and f). The activation of p53 can lead to either the promotion of apoptosis or the induction of senescence. The p21^cip^ is a cell cycle controller that is critical for determining the outcome of p53 activation because it induces cell cycle arrest, inhibits the proapoptotic activity of p53 and channels p53 activity towards the induction of senescence.^29^ After we blocked the p53/p21 pathway, the percentage of SA-*β*-Gal-positive cells was significantly lower in the leptin-+PFT-*α*-treated CPCs. In contrast, there was no obvious change in the percentage of SA-*β*-Gal-positive cells in the leptin-+P-p38 inhibitor-treated cells, although the levels of P-p38 were lower in these cells than in the CPCs that were treated with leptin alone (Figures 4b and c). These results show that leptin activates the p53/p21 pathway to induce CPCs senescence.

Sirt1 is often associated with p53-mediated senescence because it reduces levels of p53 deacetylase and stabilizes p53,^30^ which has damaging effects that reduce p53 functions. Activating Sirt1 using resveratrol demonstrated that Sirt1 signalling is involved in leptin-induced senescence in CPCs (Figures 5b and c). We also found that articular cartilage tissue sections that were derived from Sirt1 KO mice that were up to 3 months old exhibited higher levels of p53 and p21 than were expressed in the comparable sections obtained from WT mice (Figures 5e and f). Sirt1 pathway inhibition and p53/p21 pathway activation appear to have a synergistic effect on senescence in CPCs, which includes cartilage degeneration and OA.

The loss of normal functionality that results from the induction of senescence has been implicated in age-related diseases and tissue degeneration.^31, 32, 33^ By comparing cartilage tissues that were harvested from the same OA knee joint that displayed different levels of degeneration, we found that the level of leptin was homogeneous and that the impact of leptin was variable. We observed more serious degeneration in cartilage tissues (low Coll-2 expression) with higher Ob-Rb expression (which activates the leptin pathway), higher p53/p21 expression (which induces tissue senescence) and lower Sirt1 expression in the same knee joint (Figures 6a and b). Our data support the existence of a relationship between Ob-Rb and Coll-2, the p53/p21 pathway and the Sirt1 pathway (Figures 6d–f) and the notion that leptin-induced senescence is induced in CPCs via the activation of the p53/p21 pathway and the inhibition of the Sirt1 pathway. We also show that these processes may be more dependent on the function of Ob-Rb in OA patients and that they may be an important contributor to OA. These results also explain why more thinning is observed in medial tibial cartilage during cartilage degeneration,^8^ which is accompanied by an increase in the expression of Ob-Rb (Figure 6a).

BMI or body fat percentage show relative increases in some humans with increasing age, and it also appears that leptin levels tend to become inevitably elevated with age.^7^ Our results show that elevated leptin levels can have harmful effects on CPCs functions and can accelerate cartilage degeneration. Weight control may be an important means to prevent OA. However, whether leptin-induced senescence in CPCs and cartilage degeneration can be interrupted and whether the higher levels of Ob-Rb expression that were observed in severe cartilage degeneration were evoked by biomechanical forces will require further study.

## Conclusions

In summary, leptin alters the fate of differentiating CPCs by inhibiting chondrogenesis and increasing osteogenesis. Leptin induces senescence in CPCs by activating the p53/p21 pathway and inhibiting the Sirt1 pathway. The activation of the leptin pathway (higher Ob-Rb expression) is significantly correlated with cartilage degeneration (lower Coll-2 levels) and tissue senescence (higher p53/p21 levels and lower Sirt1 levels) in OA patients, suggesting that leptin-induced CPCs senescence contributes to the development of OA. Taken together, our results suggest new links between obesity and cartilage damage that involve leptin-mediated effects on CPCs differentiation and senescence.

## Materials and Methods

### OA cartilage collection

OA cartilage samples were obtained from five patients (age 55–60 years old) who underwent total knee arthroplasty for clinically and radiologically diagnosed OA. Patients were excluded if they presented with a history of rheumatic arthropathies or infection in the knee.^11^ All tissues were obtained with fully informed consent and prior institutional ethical approval. All samples were harvested from the major load-bearing areas on the MFC, LFC, MTP and LTP.

### Normal cartilage tissue harvesting and CPC isolation

Fresh joints were obtained from Sprague–Dawley rats (8 weeks old). Articular cartilage was harvested and subjected to sequential trypsin/collagenase digestion to isolate chondrocytes, as previously described.^5^ Single-cell cartilage-derived cells were cultured in 96-well plates for 7–10 days, and the colonies were then collected at the same time.^13^ The colonies were passaged two or three times prior to use in all experiments. DMEM/F12 supplemented with 10% FBS (Gibco, Grand Island, NY, USA; lot number 1652790), penicillin/streptomycin (50 000 U/50 mg) and l-glutamine (4.5 mM) was used to expand single-cell colonies that were derived from cartilage. Cells were cultured under standard conditions.

### Flow cytometry

One million CPCs (passage 3) were washed in PBS and incubated for 1 h at 4 °C with conjugated antibodies against CD90-FITC, CD29-PE, CD45-FITC, CD49e-FITC or isotype control (all from eBioscience, San Diego, CA, USA). Cells were centrifuged at 200x*g*, the supernatants were removed and the cells were washed three times in PBS. The labelled cells were resuspended in 1 ml of PBS and subjected to single channel fluorescence-activated cell sorting (FACS) analysis. The appropriate IgG controls were included, and control experiments were run in parallel.

### Immunocytochemistry

To immunostain cells or tissues (frozen sections), the samples were fixed in 4% paraformaldehyde in PBS for 10 min at room temperature. After the cells were washed three times in PBS/0.1% BSA for 5 min, they were permeabilized using 0.2% Triton (Sigma, St Louis, MO, USA; T9284) in PBS for 20 min and then washed in PBS/0.1% BSA. Primary antibodies against CD73, SOX-9, RUNX-2, Coll-2, Coll-1, Ob-Rb, p53, p21 (all Abcam, Cambridge, UK) and CD146 (sc-18942; Santa Cruz, CA, USA) were diluted in PBS/0.1% BSA to 1/150 and incubated overnight at 4 °C. After the samples were washed, the cells or frozen sections were incubated with a FITC-conjugated goat anti-rabbit secondary antibody (1:500; Abcam) and DAPI (Sigma) for 1 h at room temperature. Fluorescent images were obtained using a Nikon A1-R (Melville, NY, USA) inverted confocal microscope.

### IHC

Knee cartilage was obtained from 8-week-old rats and subjected to IHC analysis using anti-CD146 (sc-18942; Santa Cruz) antibodies. Briefly, the knee cartilage was de-paraffinized and rehydrated and then subjected to antigen retrieval by incubating the tissues in hot (95 °C) sodium citrate buffer (0.01 M, pH 6.0) for 10 min. The tissue sections were exposed to hydrogen peroxide (3% H_2_O_2_) for 5 min to quench the endogenous peroxidase and then blocked in 30% horse serum for 30 min. The slides were incubated overnight at 4 °C with primary CD146 antibodies (1:150 dilution, see above). Non-immune mouse IgG was used as a negative control. After the tissues were washed with 1 × TBST (Tris-buffered saline containing 0.1% Tween-20), the slides were then incubated with biotinylated secondary antibodies (anti-goat IgG; Santa Cruz) and detected using an ABC kit (Vector Labs, Burlingame, CA, USA).

### Cell differentiation assays

We investigated the *in vitro* multi-fate potential of the CPCs to determine whether they possessed osteogenic, adipogenic and chondrogenic potential, as previously described.^1^ Osteogenic differentiation was quantified in CPCs using Alizarin Red S staining. Adipocytes were visualized using 0.3% Oil Red O staining for adipogenesis (Sigma). Chondrogenic differentiation was assessed in CPCs by staining cells and tissues using Alcian Blue (Sigma-Aldrich), Coll-2 and Coll-1 (Abcam).

### Cell migration/chemotaxis assay

Cell migration assays were performed using a CytoSelect 24-Well Cell Invasion Assay kit according to the manufacturer's instructions.^34^ CPCs cell suspensions (10 000 cells in serum-free medium in the presence of different leptin levels (10 , 50 and 100 ng/ml)) were added to the upper well for Transwell assays. The plates were incubated for 24 h prior to processing. The migrated cells were counted in five visual fields using a microscope.

### Effects of leptin on CPC proliferation

Cells were seeded into 96-well plates at 1 × 10^4^ cells/well to measure cell viability. The cells were treated with various drugs for 48 h. Cell viability was determined using CCK-8 assays according to the manufacturer's instructions, and the results were normalized to the results in the non-treatment control group.

### Cell cycle analysis

Cells (1 × 10^6^ cells per sample) were collected and passed through a 40-*μ*m nylon membrane. Cold 75% alcohol (1 ml) was added to the cells for 24 h. Then the cells were then resuspended in 250 *μ*l of PI solution/0.1% Triton X-100/RNase A, incubated at room temperature for 30–45 min and subjected to single channel FACS analysis.

### Senescence-associated *β*-galactosidase staining

To detect senescence, CPCs were treated with different doses leptin for 48 h and fixed. They were then cultured in *β*-galactosidase staining buffer for 24 h (Cell Signaling Technology, Danvers, MA, USA). The cells were visualized using a BX40 Olympus microscope (Miami, FL, USA) equipped with a Nikon camera (Melville, NY, USA). The total number of cells and the number of *β*-galactosidase-positive cells were determined for 5 fields of view (× 100 magnification) per sample.^15^ The total number of cells was independently quantitated, and the percentage of senescent cells was calculated accordingly.

### Cell signalling studies

CPC cultures were treated with 100 ng/ml of leptin (R&D, Minneapolis, MN, USA; 598-LP-05M) and the phospho-p38 inhibitor SB203580 (Sigma) or the p53 inhibitor PFT-*α* (Selleck, Houston, TX, USA). The medium used to cultures the cells was DMEM/F12 supplemented with 5% fetal bovine serum, penicillin/streptomycin (50 000 U/50 mg) and l-glutamine (4.5 mM). After 48 h of treatment, phospho-p38 and p21 were detected using western blot analysis. CPCs cultures were treated with the p53 inhibitor PFT-*α* or the p38 inhibitor SB203580, both with or without 100 ng/ml leptin. The expression of acetyl p53 was evaluated in CPCs after the cells were treated with high doses of leptin (100 ng/ml) and resveratrol (30* μ*M). At 48 h after treatment began, cell senescence was evaluated using a commercial staining kit. SA-*β*-Gal-positive cells were counted in five visual fields using light microscopy (× 100 magnification).

### Western blot analysis

Cells were sonicated in standard lysis buffer containing protease and phosphatase inhibitors. Western blots were performed using standard protocols, and proteins were visualized using Pierce West Dura detection reagent and a Chemi Doc-It Imaging System attached to a Biochemi HR camera (Upland, CA, USA).^1^ To measure protein abundance, the grey values of the blots in the scanned images were measured using ImageJ Plus software (National Institutes of Health, Bethesda, MD, USA). Before comparisons were made, the grey value of each target protein was normalized to the value for *β*-actin or GAPDH.^35^

### Sirt1 KO mice

Sirt1 knockout mice were generated using a Cre/loxP recombination system. Mice containing a loxP-flanked Sirt1 exon (Sirt1 loxP/loxP) and an Mx promoter sequence-modified Cre recombinase gene were obtained from Jackson Laboratories (Bar Harbor, Maine, USA). Sirt1 loxP/loxP and Mx-Cre strains were mated, and homozygous Sirt1 loxP/loxP and heterozygous Sirt1 loxP/△ mice with or without MX-Cre(+/−) were identified. Sirt1 loxP/△ MX+ mice were backcrossed with Sirt1 loxP/loxP MX− mice to generate Sirt1 loxP/loxP Mx+ mice (deleted) and Mx− littermates (Sirt1 wild type). Seven-week-old mice were administered intraperitoneal injections of 400 mg of Poly I-C every 3 days for a total of three doses. Sirt1 loxP/loxP Mx+ mice that received Poly I-C treatment are referred to as Sirt1−/− mice, whereas Sirt1 loxP/loxP Mx− mice are referred to as WT mice. Female mice that were 8–14 weeks old and weighed 20–28 g were used in all studies.

### Statistical analysis

All *in vitro* experiments were repeated at least three times, and different samples were used for each experimental replicate. The results from the *in vitro* experiments were analysed using one-way analysis of variance (ANOVA) or *t*-tests if only two conditions were being compared. The data from immunohistochemistry experiments performed on mouse specimens were analysed using Student's *t*-tests. All data were analysed using Prism V.5.0b software (GraphPad Software, LaJolla, CA, USA). *P*-values ⩽0.05 were considered statistically significant. The results are expressed as the means±S.D.

## Abbreviations

CPCs: chondrogenic progenitor cells
OA: osteoarthritis
Ob-Rb: leptin receptor
RUNX-2: runt-related transcription factor 2
Coll-2: collagen II
SA-*β*-Gal: senescence associated *β*-galactosidase
Sirt: silent information regulator
